# Emotional intelligence: a comparison between patients after first episode mania and those suffering from chronic bipolar disorder type I

**DOI:** 10.1017/S0033291721005122

**Published:** 2023-05

**Authors:** Cristina Varo, Silvia Amoretti, Giulio Sparacino, Esther Jiménez, Brisa Solé, Caterina del Mar Bonnin, Laura Montejo, Maria Serra, Carla Torrent, Estela Salagre, Antoni Benabarre, Pilar Salgado-Pineda, Irene Montoro Salvatierra, Pilar A. Sáiz, María Paz García-Portilla, Vanessa Sánchez-Gistau, Edith Pomarol-Clotet, Josep Antoni Ramos-Quiroga, Isabella Pacchiarotti, Clemente Garcia-Rizo, Juan Undurraga, María Reinares, Anabel Martinez-Aran, Eduard Vieta, Norma Verdolini

**Affiliations:** 1 Bipolar and Depressive Disorders Unit, Institute of Neuroscience, Hospital Clinic, University of Barcelona, IDIBAPS, CIBERSAM, 170 Villarroel st, 12-0, 08036, Barcelona, Spain; 2 Biomedical Research Networking Center for Mental Health Network (CIBERSAM), Barcelona, Spain; 3 Barcelona Clinic Schizophrenia Unit, Institute of Neurosciences, University of Barcelona, IDIBAPS, CIBERSAM, Barcelona, Catalonia, Spain; 4 Psychiatric Genetics Unit, Vall d'Hebron Research Institute (VHIR), Barcelona, Catalonia, Spain; 5 Department of Psychiatry, Hospital Universitari Vall d'Hebron, Barcelona, Catalonia, Spain; 6 Department of Health Sciences, Università degli Studi di Milano, Milan, Italy; 7 FIDMAG Germanes Hospitalàries Research Foundation, c/Dr. Pujades 38, 08830, Sant Boi de Llobregat, Barcelona, Spain; 8 Hospital Universitari Institut Pere Mata, Institut d'Investigació Sanitària Pere Virgili (IISPV), Universitat Rovira i Virgili, CIBERSAM, Reus, Tarragona, Spain; 9 Deparment of Psychiatry, University of Oviedo, Instituto de Investigación Sanitaria del Principado de Asturias, ISPA, Mental Health Services of Principado de Asturias, SESPA, Oviedo, Spain; 10 Department of Psychiatry and Legal Medicine, Universitat Autònoma de Barcelona, Barcelona, Spain; 11 Department of Neurology and Psychiatry, Faculty of Medicine, Clinica Alemana Universidad del Desarrollo, Santiago, Chile; 12 Early Intervention Program, Instituto Psiquiátrico Dr. J. Horwitz Barak, Santiago, Chile

**Keywords:** Bipolar disorder, emotional intelligence, first episode mania, MSCEIT, verbal memory

## Abstract

**Background:**

Deficits in emotional intelligence (EI) were detected in patients with bipolar disorder (BD), but little is known about whether these deficits are already present in patients after presenting a first episode mania (FEM). We sought (i) to compare EI in patients after a FEM, chronic BD and healthy controls (HC); (ii) to examine the effect exerted on EI by socio-demographic, clinical and neurocognitive variables in FEM patients.

**Methods:**

The Emotional Intelligence Quotient (EIQ) was calculated with the Mayer-Salovey-Caruso Emotional Intelligence Test (MSCEIT). Performance on MSCEIT was compared among the three groups using generalized linear models. In patients after a FEM, the influence of socio-demographic, clinical and neurocognitive variables on the EIQ was examined using a linear regression model.

**Results:**

In total, 184 subjects were included (FEM *n* = 48, euthymic chronic BD type I *n* = 75, HC *n* = 61). BD patients performed significantly worse than HC on the EIQ [mean difference (MD) = 10.09, standard error (s.e.) = 3.14, *p* = 0.004] and on the understanding emotions branch (MD = 7.46, s.e. = 2.53, *p* = 0.010). FEM patients did not differ from HC and BD on other measures of MSCEIT. In patients after a FEM, EIQ was positively associated with female sex (*β* = −0.293, *p* = 0.034) and verbal memory performance (*β* = 0.374, *p* = 0.008). FEM patients performed worse than HC but better than BD on few neurocognitive domains.

**Conclusions:**

Patients after a FEM showed preserved EI, while patients in later stages of BD presented lower EIQ, suggesting that impairments in EI might result from the burden of disease and neurocognitive decline, associated with the chronicity of the illness.

## Introduction

Neurocognitive impairment is a well-established feature in bipolar disorder (BD), even in the early stages of disease (Pope, Mazmanian, & Sharma, 2016). It is present also in many cases during euthymic periods and is an important determinant of psychosocial functioning (Pope et al., 2016). Although neurocognition has been more exhaustively studied, over the past decades there has been an increased interest in the study of social cognition (SC) (Varo et al., 2019, 2020) which is defined as the ability to detect, process, and use social information to manage interpersonal functioning and social behavior. SC deficits may produce significant daily difficulties given the crucial importance of SC for social relations and well-being (Miskowiak & Varo, 2021). SC encompasses five distinct areas, namely (i) emotional processing, (ii) theory of mind, (iii) attributional bias, (iv) social perception, (v) social knowledge (Green, Horan, & Lee, 2019). In BD research, the study of SC has focused mainly on emotional processing, which has been also conceptualized as emotional intelligence (EI) (Samamé, Martino, & Strejilevich, 2015), and generally measured by means of the Mayer-Salovey-Caruso Emotional Intelligence Test (MSCEIT) (Mayer, Salovey, Caruso, & Sitarenios, 2003).

Deficits in EI have been detected in patients with chronic BD (Aparicio et al., 2017; Frajo-Apor et al., 2020; McClure et al., 2005; Samamé et al., 2015; Varo et al., 2019, 2020). However, the evolution of EI throughout the course of BD is unclear due to the paucity of studies that have examined the deficits in EI in patients experiencing a first episode mania (FEM) (Daros, Ruocco, Reilly, Harris, & Sweeney, 2014; Szmulewicz, Lomastro, Valerio, Igoa, & Martino, 2019) and the lack of longitudinal studies on EI of these patients. It remains to be solved whether the deficits are present since the beginning of the disease (i.e. as primary deficits) and remain stable from early stages to chronicity, or whether they emerge and worsen as a result of the burden of disease related with the chronicity of the illness (i.e. as secondary deficits). Moreover, to the best of our knowledge, no study so far has assessed EI in FEM patients in comparison with those in later stages of BD.

Previous evidence for the role of EI for patients suffering from a non-affective first episode psychosis (FEP) has been reported (Sanchez–Gistau et al., 2020). EI was found to be altered in non-affective FEP patients at onset and its impairment represents a stable pattern and a relevant feature of early schizophrenia (Green et al., 2012). Schizophrenia and BD share a chronic clinical course with impairments in neurocognitive and clinical features, although with different levels of severity (Lee et al., 2013). As a consequence, patients with a FEM might present a similar but subtler pattern of EI abnormalities than non-affective FEP patients. To date, no study has investigated the association between socio-demographic, clinical, neuropsychological variables and EI among patients with a FEM. A better comprehension of the relationship between these variables and EI performance would have implications in understanding the nature, trajectory, and clinical relevance of the difficulties on this SC domain in the early stages of BD. Considering these gaps in the literature, the main aim of the present study was to explore EI using the full version of the MSCEIT in patients after a FEM in comparison with patients with chronic BD and healthy controls (HC). Also, the secondary aim was provided insight on the potential contribution of socio-demographic, clinical, and neurocognitive variables on EI performance in patients after a FEM. We hypothesized that FEM patients would present intermediate EI performance between HC and chronic BD, and their performance would be influenced by neurocognitive performance, clinical and socio-demographic variables.

## Material and methods

### Participants

Data were pooled from two projects developed by our research group. The first project recruited FEM patients as part of a 2-year longitudinal multicentric study including the Bipolar and Depressive disorders Unit of IDIBAPS-Hospital Clinic in Barcelona, FIDMAG Research Foundation, and the University Hospital Institut Pere Mata. The second project recruited cross-sectionally chronic BD patients both at the Hospital Clinic in Barcelona and at mental health services in Oviedo. HC were recruited through advertisement at the Hospital Clinic in Barcelona. The four centers cooperate under the umbrella of the Spanish Research Network on Mental Health (CIBERSAM) (Salagre et al., 2019).

The inclusion criteria for FEM patients, evaluated at baseline, were: (i) aged between 18 and 45 years old at the time of first evaluation; (ii) having experienced their FEM (with or without psychotic symptoms) over the previous 3 years; (iii) being in full or partial remission [Hamilton Depression Rating Scale 17-item (HDRS-17) (Hamilton, 1960; Ramos-Brieva & Cordero-Villafafila, 1988) ⩽14 and Young Mania Rating Scale (YMRS) (Colom et al., 2002; Young, Biggs, Ziegler, & Meyer, 1978) ⩽14]. The inclusion criteria for patients with BD were: (i) aged over 18 years old; (ii) fulfilling DSM-IV-TR criteria for BD type I (BD-I) and (iii) being euthymic (HDRS-17⩽8, YMRS⩽6), at least in the 3 months before the inclusion. Patients could have experienced more than one affective episode over the previous 3 years, could then be considered within their early-stage BD illness.

Exclusion criteria for both FEM and BD patients were the presence of (i) a mental intellectual disability [defined as intelligence quotient (IQ) <70]; (ii) presence of any medical condition affecting neuropsychological performance; (iii) alcohol/substance dependence in the previous year to study inclusion; (iv) having received electroconvulsive therapy in the 12 months before participation.

All patients were under stable treatment regimen.

HC without current or past psychiatric history, meeting the same exclusion criteria as patients, were recruited via advertisement. In addition, HC were asked if they had first-degree relatives with psychiatric disorders.

The study was carried out following the latest version of the Declaration of Helsinki, and it was reviewed by the ethical committee of the four institutions. Written informed consent was obtained from all participants.

### Clinical assessment

In order to gather clinical data, all patients were assessed by means of the Structured Clinical Interview for DSM Disorders (SCID-I-II) (First, Gibbon, Spitzer, Williams, & Benjamin, 1997*a*, 1997*b*). The YMRS and HDRS-17 scores were used to evaluate the severity of manic and depressive symptomatology, respectively. All the participants also completed the Functional Assessment Short Test (FAST) (Rosa et al., 2007), a scale designed to assess psychosocial functional impairment in psychiatric patients, with higher scores indicating poorer psychosocial functioning. The full description of other clinical variables is reported in the online Supplementary Material.

### Emotional intelligence assessment

EI was evaluated using the Spanish version of the MSCEIT, V2.0 (Mayer et al., 2003). This instrument consists of 141 items and provides eight task scores that measure the four branches of EI: (i) perceiving emotions: to recognize and to appraise emotions accurately; (ii) using emotions: to access or generate feelings when they facilitate thoughts; (iii) understanding emotions: to understand complex emotions and how emotions transition from one stage to another, to recognize the causes of emotions, and to understand relationships among emotions; (iv) managing emotions: to stay aware of one's emotions, and to solve emotion-laden problems. The perceiving emotions and using emotions branches are assigned to the experiential area, while the understanding emotions and managing emotions branches are assigned to the strategic area. The test provides an overall score, the EI Quotient (EIQ), and also scores in the two areas, in the four branches and in each of the specific tasks. Lower scores indicate poorer performance in EI. The average range of EIQ is 100, with a standard deviation (s.d.) of 15.

### Neuropsychological assessment

All participants were evaluated using a comprehensive neuropsychological battery exploring different cognitive domains: processing speed, working memory, verbal learning and memory, visual memory, executive functions and attention. The neuropsychological battery comprised the digit-symbol coding, symbol search, arithmetic, digits, and letter-number sequencing subtests from Wechsler Adult Intelligence Scale (WAIS-III) (Wechsler, 1997), phonemic (F-A-S) and categorical (animal naming) components of the Controlled Oral Word Association Test (COWAT) (Patterson, 2018), the Trail Making Test-A (TMT-A) and Trail Making Test-B (TMT-B) (Reitan, 1958), the California Verbal Learning Test (CVLT) (Delis, Kramer, Kaplan, & Over, 1987), the Rey Osterrieth Complex Figure (ROCF) (Rey, 2009), the computerized version of the Wisconsin Card Sorting Test (WCST) (Heaton, Chelune, Talley, Kay, & Curtiss, 1993), the Stroop Color-Word Interference Test (Golden, 1994), and the Continuous Performance Test-II (CPT-II), version 5 (Conners, 2002). Finally, estimated IQ was assessed with the (WAIS-III) vocabulary subtest (Wechsler, 1997).

### Statistical analysis

Comparison of socio-demographic and clinical characteristics among groups (FEM, BD, and HC) was carried out using χ^2^ tests for categorical variables and analysis of variance for continuous variables. The Tukey's test was carried out for post-hoc comparisons to identify pair-wise differences between groups. Effect sizes (Glass's *d*) were also calculated to estimate the magnitude of the differences between the groups. Neurocognitive tests raw scores were standardized to *z*-scores based on HCs' performance (for further information on the calculation of the composites of neurocognitive domains, see Supplementary Material). Performance on MSCEIT and the neurocognitive domains was compared across the three groups using generalized linear models. All models were adjusted for those clinical and socio-demographic variables for which the three groups differed significantly. Then, a Bonferroni post-hoc correction was applied when significant main effects were present when comparing the three groups, in order to identify pair-wise differences between groups. Estimated marginal means, adjusted for the other variables in the model, were reported for each variable of interest (i.e. EIQ), as well as the 95% confidence interval (CI), their mean difference (MD) and its standard error (s.e.).

Moreover, exploratory analyses were conducted to satisfy our secondary aim. In order to assess which socio-demographic, clinical, and neuropsychological variables were associated with IEQ in the FEM and in the BD groups, we first performed Pearson bivariate correlations to identify those continuous variables significantly associated with EIQ. For categorical variables (i.e. sex), Student's *t* test was run to evaluate the distribution of EIQ. Only those variables with a *p* value ⩽0.05 were then entered into a hierarchical multiple regression model, aimed at evaluating the association between socio-demographic, clinical, and neuropsychological variables and EIQ.

All statistical analyses were conducted using IBM SPSS Statistics version 23.0. Statistical significance was set at *p* < 0.05.

## Results

The total sample included 184 participants: 48 patients with a FEM in full or partial clinical remission, 75 euthymic BD patients and 61 HC. Socio-demographic variables among groups are reported in Table 1.

**Table 1.** Socio-demographic and clinical variables of first episode mania (FEM) or bipolar disorder (BD) patients and healthy controls (HC)

Variables		FEM (A) (*n* = 48, 26.09%)	BD (B) (*n* = 75, 40.76%)	HC (C) (*n* = 61, 33.15%)	Statistics				
					χ^2^ or *F*	*p*	Pairwise comparison Tukey HSD or χ^2^*	*p*	Effect size (Glass's delta)
*Socio-demographic variables*									
Age	Mean (s.d.)	28.31 (7.40)	45.87 (10.53)	38.72 (11.09)	44.970	**<0.001**	B < A < C	**<0**.**001** **<0**.**001** **<0**.**001**	1.40 0.68 2.37
Sex (women yes)	*n* (%)	25 (52.1)	45 (60.0)	37 (60.7)	0.989	0.637			
Civil status (married yes)	*n* (%)	12 (25.0)	28 (37.3)	28 (46.7)	5.483	0.068	A < C	**0**.**027**	
Education level	*n* (%)				8.990	**0.011**			
Secondary school		26 (54.2)	43 (57.3)	20 (32.8)			A > C B > C	**0**.**041** **0.006**	
University		22 (45.8)	32 (42.7)	41 (67.2)			A < C B < C	**0**.**041** **0.006**	
Employment	*n* (%)				62.335	**<0.001**			
Studying		16 (33.3)	4 (5.3)	6 (9.8)			B < A C < A	**<0**.**001** **0.005**	
Working		15 (31.3)	24 (32.0)	49 (80.3)			A < C B < C	**<0**.**001** **<0.001**	
Not studying/not working		17 (35.4)	47 (62.7)	6 (9.8)			A < B C < A C < B	**0**.**003** **0.006** **<0.001**	
Estimated IQ	Mean (s.d.)	105.13 (11.96)	106.12 (15.70)	109.75 (9.89)	2.008	0.137			
*Clinical variables*									
Family history of BD	*n* (%)	12 (25.0)	17 (23.3)	–	<0.001	1.000			
Family history of MDE	*n* (%)	18 (37.5)	26 (35.6)	–	<0.001	0.986			
Age at onset	Mean (s.d.)	24.15 (8.40)	25.21 (8.94)	–	0.049	0.825			
Onset polarity	*n* (%)				2.562	0.265			
Mania		25 (52.1)	30 (40.0)	–					
Depression		20 (41.7)	42 (56.0)	–					
Hypomania		3 (6.3)	3 (4.0)	–					
Age at first hospitalization^a^	Mean (s.d.)	27.57 (7.58)	31.20 (11.20)	–	7.184	**0.009**			**0.48**
Duration of illness	Mean (s.d.)	4.17 (5.01)	20.65 (8.98)	–	13.058	**<0.001**			**3.29**
Number of episodes	Mean (s.d.)								
Total		2.35 (1.28)	10.41 (8.55)	–	19.480	**<0.001**			**6.29**
Mania		1.19 (0.53)	3.62 (4.00)		19.969	**<0.001**			**4.62**
Hipomania		0.23 (0.59)	1.86 (3.23)		19.435	**<0.001**			**2.76**
Depression		0.88 (0.98)	4.45 (4.31)		21.127	**<0.001**			**3.64**
Mixed episodes		0.06 (0.24)	0.46 (1.4)		13.351	**<0.001**			**1.67**
Psychiatric comorbidities	*n* (%)								
Axis I		4 (8.3)	17 (23.0)	–	3.412	0.065			
Axis II		4 (8.3)	15 (20.3)	–	2.313	0.128			
Axis III		11 (22.9)	19 (26.0)	–	0.030	0.863			
FAST total score^b^	Mean (s.d.) [range]	16.79 (13.16) [1–64]	25.53 (14.45) [0–61]	5.27 (4.48) [0–20]	49.449	**<0.001**	B < A<C	**<0**.**001** **<0.001** **<0.001**	0.87 4.45 0.64
YMRS total score^b^	Mean (s.d.) [range]	1.10 (0.63) [0–7]	1.68 (1.63) [0–6]	0.63 (1.01) [0–3]	8.556	**<0.001**	C < B	**<0**.**001**	1.01 0.87
HAM-D total score^b^	Mean (s.d.) [range]	4.15 (2.94) [0–10]	4.07 (2.52) [0–8]	1.67 (1.78) [0–6]	20.173	**<0.001**	C < A C < B	**<0**.**001** **<0.001**	0.84 0.91
Psychotropic medication^b^	*n* (%)								
Lithium		38 (79.2)	50 (66.7)	–	1.674	0.196			
Antiepileptic		8 (16.7)	38 (50.7)	–	15.404	**<0.001**			
Antipsychotic		25 (52.1)	59 (78.7)	–	8.364	**0.004**			
Antidepressant		4 (8.3)	28 (37.3)	–	11.326	**0.001**			
Benzodiazepines		7 (14.6)	13 (17.3)	–	0.023	0.879			

BD, bipolar disorder; FAST, Functioning Assessment Short Test; HAM-D, Hamilton Depression Rating Scale; IQ, intelligence quotient; MDE, major depressive episode; s.d., standard deviation; YMRS, Young Mania Rating Scale.

*Only statistically significant or almost significant comparisons are reported. Bold for statistically significant values.

a Missing information for seven FEM. Four FEM and 14 BD patients had no history of hospitalization.

b At time of evaluation.

### Clinical features among the groups

Regarding clinical variables, there were significant differences between patient groups (FEM and chronic BD) and HC in the total HDRS-17 (*p* < 0.001) and YMRS scores (*p* < 0.001), as well as in the overall psychosocial functioning (*p* < 0.001). Both patient groups presented more subsyndromal depressive symptoms than HC (BD *v.* HC *p* < 0.001, FEM *v.* HC *p* < 0.001, respectively), whereas chronic BD patients exhibited more subsyndromal manic symptoms than HC (*p* < 0.001). No statistically significant differences were found in subsyndromal symptoms between patient groups. Significant group differences in the FAST total score were observed for both the patient groups, presenting significantly decreased functioning compared to HC (*p* < 0.001). In addition, chronic BD patients showed poorer psychosocial function than patients in the FEM group (*p* < 0.001).

Significant differences were observed in the comparison between chronic BD and FEM patients in age at first hospitalization (*p* = 0.009), being lower in the case of the FEM group (*p* = 0.009), but not regarding the polarity at onset (*p* = 0.265) or the presence of family history for either BD (*p* = 1.000) or major depressive disorder (*p* = 0.986). Groups differed in terms of duration of illness (*p* < 0.001) and total number of episodes (*p* < 0.001). Patients after a FEM experienced an average of 1.19 episodes of mania whilst BD chronic patients an average of 3.62.

### Emotional intelligence performance

**Fig. 1.**
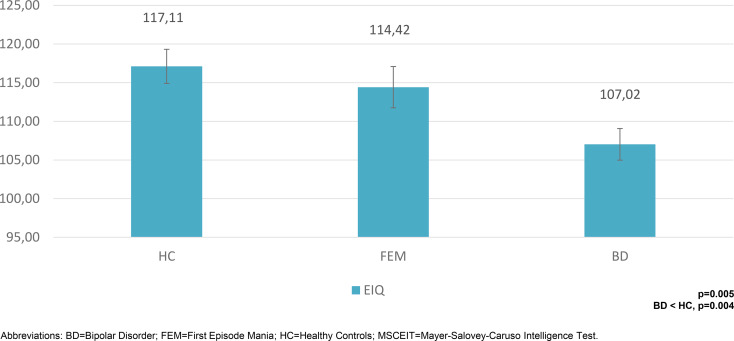
Emotional intelligence quotient with error bars in the three groups.BD, bipolar disorder; FEM, first episode mania; HC, healthy controls; MSCEIT, Mayer-Salovey-Caruso Intelligence Test.

**Fig. 2.**
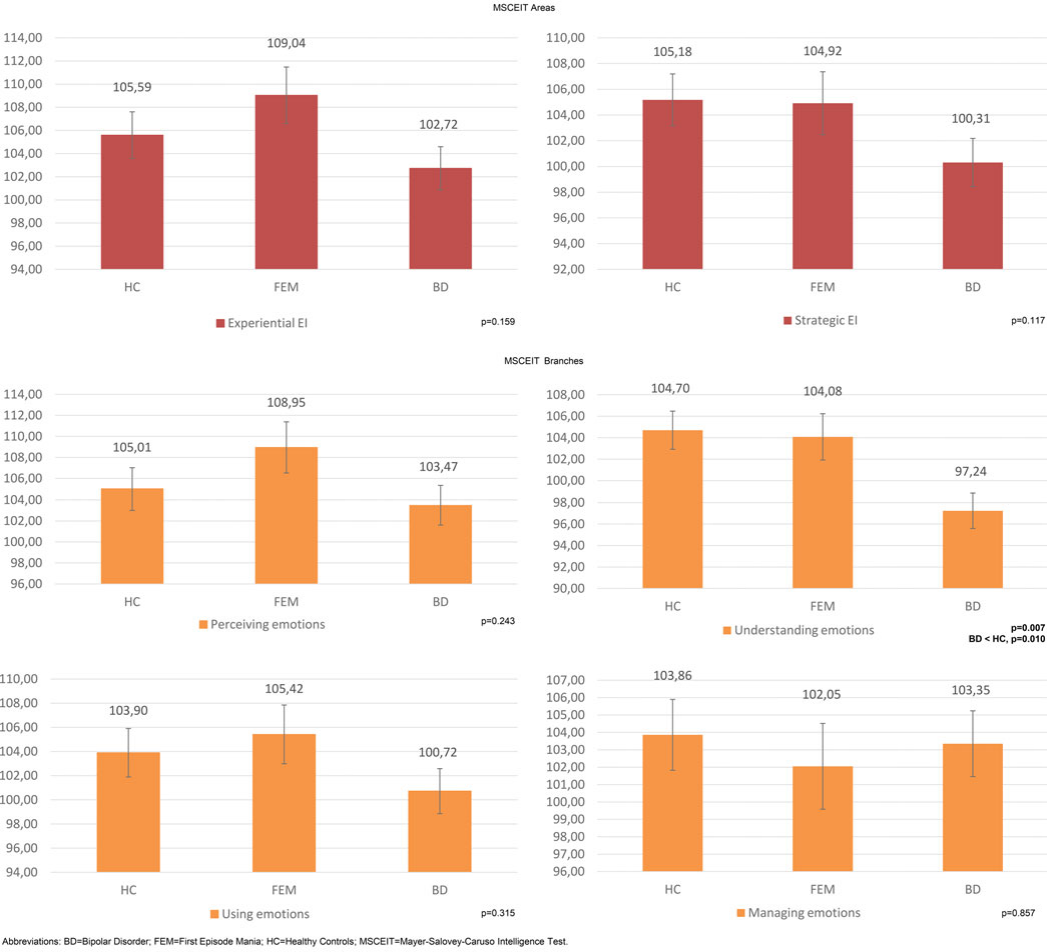
Mean MSCEIT scores with error bars in the three groups. BD, bipolar disorder; FEM, first episode mania; HC, healthy controls; MSCEIT, Mayer-Salovey-Caruso Intelligence Test.

Patients in the FEM group performed similarly to HC on MSCEIT Total score (online Supplementary Table S1, Fig. 1) and all measures of MSCEIT (online Supplementary Table S1, Fig. 2).

Significant differences were found for EIQ (*p* = 0.005) and in the MSCEIT understanding emotions branch (*p* = 0.007), even after controlling for age, subsyndromal manic and depressive symptoms. Bonferroni post-hoc testing revealed that BD patients presented significantly lower EIQ than HC (MD = 10.09, s.e. = 3.14, *p* = 0.004) but no difference was found neither between HC and FEM patients (MD = 2.69, s.e. = 3.56, *p* = 1.000) nor between FEM and chronic BD patients (MD = 7.40, s.e. = 3.61, *p* = 0.121).

In addition, BD patients performed more poorly than HC on the understanding emotions branch (MD = 7.46, s.e. = 2.53, *p* = 0.010). A trend-level difference was reported between patient groups, with BD patients showing lower scores than those in the FEM group (MD = −6.84, s.e. = 2.93, *p* = 0.056). No significant difference was reported between FEM patients and HC (MD = 0.62, s.e. = 2.87, *p* = 1.000).

### Neurocognitive performance

**Fig. 3.**
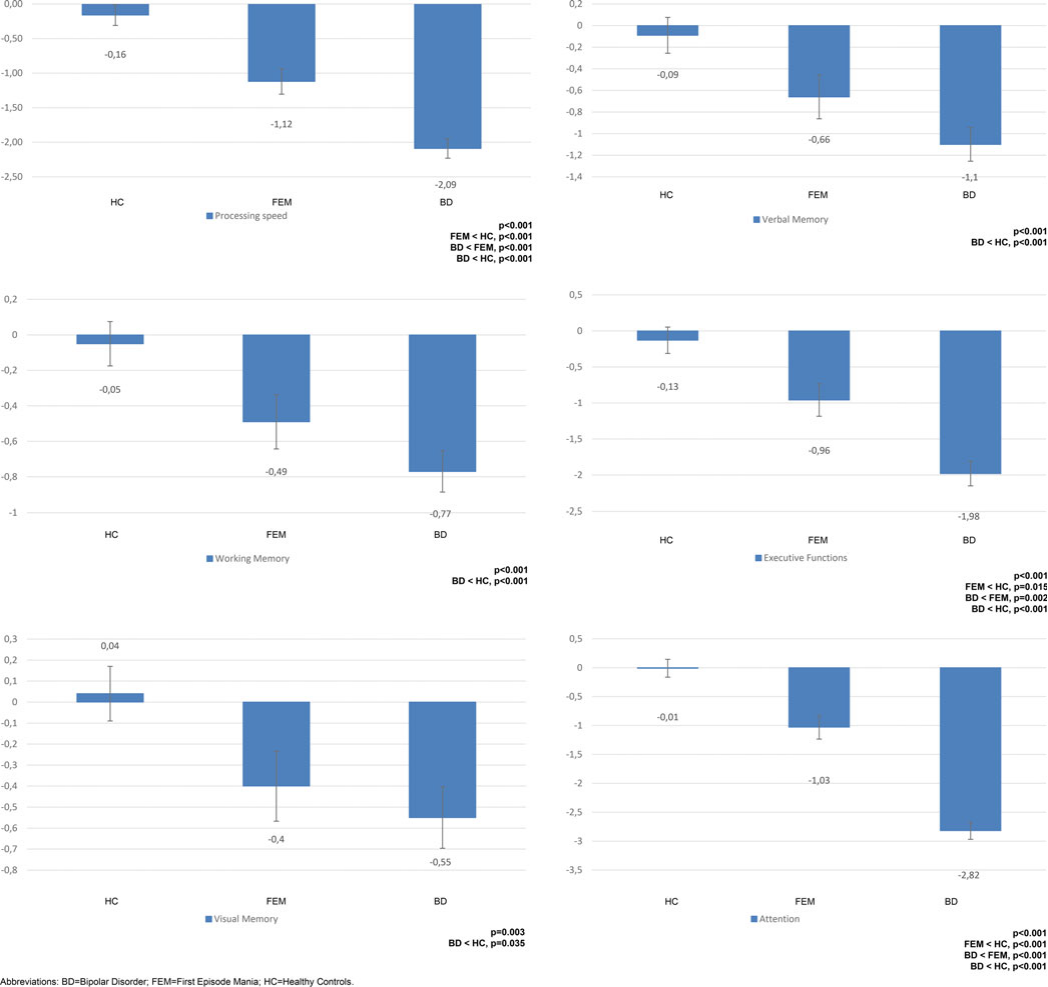
Neuropsychological composite mean scores with error bars in the three groups. BD, bipolar disorder; FEM, first episode mania; HC, healthy controls; PS, processing speed composite; VM, verbal memory composite; WM, working memory composite; EF, executive functions composite; VisM, visual memory composite; AT, attention composite.

Concerning neurocognitive domains, there was a main effect of group in terms of processing speed (*p* < 0.001), verbal memory (*p* < 0.001), working memory (*p* < 0.001), executive functions (*p* < 0.001), visual memory (*p* = 0.033), and attention (*p* < 0.001), after controlling for age, subsyndromal depressive and manic symptoms (online Supplementary Table S1, Fig. 3).

Bonferroni post-hoc pair-wise comparisons between groups revealed that FEM patients performed worse than HC on processing speed (MD = 0.96, s.e. = 0.24, *p* < 0.001), executive functions (MD = 0.83, s.e. = 0.30, *p* = 0.015), and attention (MD = 1.02, s.e. = 0.26, *p* < 0.001), but not on verbal, working, and visual memory. On the contrary, FEM patients performed better than chronic BD patients on processing speed (MD = 0.97, s.e. = 0.25, *p* < 0.001), executive functions (MD = 1.02, s.e. = 0.30, *p* = 0.002), and attention (MD = 1.79, s.e. = 0.28, *p* < 0.001), but not on verbal, working, and visual memory. Chronic BD patients performed significantly worse than HC on all neurocognitive domains: processing speed (MD = 1.93, s.e. = 0.22, *p* < 0.001), verbal memory (MD = 1.00, s.e. = 0.24, *p* < 0.001), working memory (MD = 0.72, s.e. = 0.18, *p* < 0.001), executive functions (MD = 1.85, s.e. = 0.26, *p* < 0.001), visual memory (MD = 0.51, s.e. = 0.20, *p* = 0.035), and attention (MD = 2.81, s.e. = 0.21, *p* < 0.001).

### Socio-demographic, clinical, and neurocognitive variables associated with EIQ in FEM patients

In FEM patients, lower EIQ correlated with poorer performance in verbal memory (*r* = 0.371, *p* = 0.011). Also, male patients showed lower scores in EIQ than females (*t* = 2.054, *p* = 0.046) (see Table 2). No other clinical variable correlated with EIQ.

**Table 2.** Correlations between MSCEIT Emotional Intelligence Quotient (EIQ) and socio-demographic and clinical variables in first episode mania (FEM) patients

Categorical variables	MSCEIT EIQ			
		Mean (s.d.)	Statistics	
			Student *t*	*p*
Sex	M	107.78 (17.68)	2.054	**0.046**
	F	117.64 (15.56)		
Family history of BD	Y	112.75 (16.91)	0.038	0.970
	N	112.97 (17.49)		
Family history of MDE	Y	113.61 (19.68)	−0.215	0.831
	N	112.50 (15.81)		
Psychotic symptoms at onset	Y	113.00 (17.12)	−0.110	0.913
	N	112.00 (20.42)		
Cannabis use in the prodromal phase	Y	116.96 (14.34)	−1.195	0.238
	N	111.55 (16.31)		
Alcohol use in the prodromal phase	Y	111.03 (17.39)	1.611	0.114
	N	119.50 (14.03)		
Lithium	Y	112.24 (18.55)	0.720	0.478
	N	115.50 (10.71)		
Antiepileptics	Y	114.25 (9.97)	−0.350	0.730
	N	112.65 (18.35)		
Antipsychotics	Y	114.25 (15.16)	−0.534	0.596
	N	111.58 (19.20)		
Antidepressants	Y	114.50 (27.04)	−0.191	0.850
	N	112.77 (16.46)		
Benzodiazepines	Y	116.43 (24.54)	−0.581	0.564
	N	112.32 (15.92)		
Continuous variables			Pearson correlation	*p*
Age			0.181	0.219
Estimated IQ			0.181	0.222
PAS			0.070	0.638
Duration of illness			0.172	0.242
Total number of episodes			−0.138	0.349
Number of psychiatric hospitalizations			−0.096	0.520
Age at first hospitalization			0.300	0.071
HAM-D total score^a^			−0.061	0.686
YMRS total score^a^			−0.262	0.072
FAST total score^a^			−0.038	0.796
Processing speed composite			0.111	0.457
Verbal memory composite			0.371	**0.011**
Working memory composite			−0.055	0.713
Executive functions composite			0.136	0.367
Visual memory composite			−0.008	0.961
Attention composite			0.059	0.705

BD, bipolar disorder; EIQ, Emotional Intelligence Quotient; FAST, Functioning Assessment Short Test; HAM-D, Hamilton Depression Rating Scale; IQ, intelligence quotient; MDE, major depressive episode; PAS, Premorbid Adjustment Scale; s.d., standard deviation; YMRS, Young Mania Rating Scale.

Bold for statistically significant values.

a At time of evaluation.

After including the variables significant in bivariate analyses in a hierarchical regression model [*F*_(2,43)_ = 6.202, adjusted *R*^2^ = 0.188, *p* = 0.004], both male sex (*β* = −0.293, *p* = 0.034) and the verbal memory domain (*β* = 0.374, *p* = 0.008) were significantly associated with EIQ, with a higher effect exerted by verbal memory performance.

Results for the chronic BD groups are reported in online Supplementary Tables S3 and S4.

## Discussion

To the best of our knowledge, this is the first study to comprehensively assess EI in patients after a FEM using the full MSCEIT version. The present study of EIQ in fully or partially remitted FEM (*n* = 48) *v.* chronic BD-I (*n* = 75) and HC (*n* = 61) showed three main findings. While patients after a FEM presented intermediate EIQ scores between HC and chronic BD, with EIQ scores significantly lower in BD than HC, in the MSCEIT branches, FEM patients' performance was globally comparable to HC. In addition, lower performance in understanding emotions branch was found for chronic BD patients in comparison with HC. Whilst EI appeared to be preserved in FEM patients, neurocognition, and particularly processing speed, attention, and executive functions performance was already impaired at the early stages of the illness. Lower EIQ in FEM was associated with male sex and lower performance in verbal memory.

Although EI has been widely studied in patients in later stages of BD (Aparicio et al., 2017; Frajo-Apor et al., 2020; Samamé et al., 2015; Varo et al., 2019), little is known about the EI performance of patients after a FEM and the course of EI impairment across the clinical stages of BD and the evidence is seldom conflicting. So far, only two studies assessed some level of EI patients after a FEM (Daros et al., 2014; Szmulewicz et al., 2019). Nonetheless, these studies were characterized by small sample size, which limited the generalizability of results, and only evaluated the lower levels of EI abilities such as labeling, discrimination, and appraising emotions. Daros et al. assessed 24 non-affective FEP and 16 FEM patients in comparison with 35 HC both during acute psychosis and after 7 weeks of treatment (Daros et al., 2014). Both groups of patients presented difficulties recognizing facial expressions that did not resolve with treatment and clinical stabilization. In a small sample of 26 FEM patients, Szmulewicz et al. found that in comparison with HC, FEM patients presented a compromised cognitive theory of mind performance characterized by a reduced ability to infer intentions from others whilst the affective theory of mind performance was preserved, indicating that FEM patients were capable to detect other's emotions and feelings (Szmulewicz et al., 2019). In the present study, FEM patients, in comparison with HC, did not present difficulties in EI, assessed through the full version of MSCEIT, which evaluates both lower and higher EI abilities.

Although EI appeared to be overall preserved among the patients after a FEM assessed in our study, their neurocognitive performance on processing speed, attention, and executive functions was mildly impaired. These findings are in line with a recent study assessing cognitive groups of patients after recovery from a FEM (Chakrabarty et al., 2021). The authors identified that almost the 50% of FEM patients reported selective cognitive impairment after recovery, with pronounced deficits in processing speed and lower performance in verbal memory, working memory, and executive functioning in comparison with HC. Furthermore, in line with our results, these deficits seemed to be stable over time in those patients that experienced a recurrence. Particularly, Kozicky et al. (2014) found that this impairment in cognitive performance was mostly evident in those who experienced longer manic or hypomanic episodes (Kozicky et al., 2014).

Patients suffering from chronic BD, included in this study, presented impairment in all the cognitive domains and lower EIQ and difficulties in the MSCEIT understanding emotions branch. Our results are in line with previous studies, supporting the presence of less severe impairment in SC compared to neurocognitive domains in patients with BD (Bilderbeck et al., 2016). Deficits of EI were not observed in FEM patients. This might suggest that more severe SC deficits might be associated with other conditions, such as schizophrenia, instead of BD since in non-affective FEP patients EI impairment was found to start early in the course of illness and to remain stable (Green et al., 2012). Given that EI is more severely affected in psychosis than in mania, one may argue that patients reporting psychotic symptoms during the first episode of mania might show greater difficulties in EI than patients without psychotic symptoms. Despite this, we did not find any difference in terms of EIQ between FEM patients who presented psychotic symptoms at onset and those who did not. Our findings suggest that neurocognition seemed to be already altered at the first symptomatic manic presentation, whilst EI started out intact in the FEM patients and then slightly worsened with illness course. One recurring question is whether neurocognition and SC in BD are sufficiently distinct to be considered separately. Previous studies investigating the relationship between neurocognition and EI have yielded mixed and inconclusive results. While there are studies that reported that lower levels of EI may be mediated by neurocognitive abilities (Aparicio et al., 2017; Frajo-Apor et al., 2017), others have not found a relationship between the two constructs (Fanning, Bell, & Fiszdon, 2012). Our results highlight the connection between EI and neurocognition and the idea that they are two complementary but separated constructs (DeTore, Mueser, & McGurk, 2018), with partial overlap and with a different degree of impairment. Thus, our findings were in line with many other works supporting the idea that neurocognitive ability may represent a ‘necessary, but not sufficient’ prerequisite for social cognitive abilities, especially in those that contain an emotional component (Bora, Veznedaroğlu, & Vahip, 2016; Lee et al., 2013; Varo et al., 2019). This view is consistent with studies from neuroimaging in social neuroscience (Mitchell, 2008). Nonetheless, the role of neurocognitive impairments on SC and EI in euthymic BD patients remains somewhat unclear. Therefore, the nature of this association should be the focus of further investigation.

Whilst in the present study the two groups of patients did not differ in terms of severity of symptoms at the time of evaluation, BD group performed worse than FEM group in measures of indicators assessing the burden of disease, such as longer duration of illness and higher total number of lifetime episodes, psychosocial functioning, and in the neurocognitive performance. Thus, our findings support the hypotheses that EI difficulties might be a result of the burden of disease and neurocognitive decline associated with the chronicity of the illness.

As for the socio-demographic, neurocognitive, and clinical variables associated with EIQ in patients after a FEM, lower EIQ scores were found to be associated with male sex and lower verbal memory performance. Regarding sex differences in EI, our findings are in line with previous studies in which men performed worse than women on EI in non-clinical samples (Pardeller, Frajo-Apor, Kemmler, & Hofer, 2017) and BD patients (Varo et al., 2019). As for the role played by verbal memory in EI, our finding is in line with previous literature underlining how EI performance might be associated with cognitive abilities (Eack et al., 2010; Frajo-Apor et al., 2020; Varo et al., 2019). In a previous study assessing BD patients, all neurocognitive domains were associated with EI (Varo et al., 2019). However, to date, it is difficult to ascertain which neuropsychological domain (among verbal memory, executive functions, psychomotor speed, working memory and attention) has a greater influence on SC, especially on EI. In the current study, verbal memory resulted to be the central domain involved in EI ability. EI was assessed by MSCEIT which demands an accurate interpretation of the semantic meaning of the social situation. It involves exercises related to verbal memory skills, such as association, categorization, and mental imagery. In another study assessing EI and cognitive abilities in healthy adults, verbal fluency was the only cognitive domain associated with EIQ (Pardeller et al., 2017).

In the present study, being men with worse performance in verbal memory arose as risk factors for worse EI ability. In consequence, an exhaustive assessment of SC and EI in this population would be recommended in order to tailor specific early intervention strategies (Vieta et al., 2018).

The findings of the present study should be interpreted in light of the following limitations. First, since our study used data from two separate projects, the groups were not matched and there were uneven sample sizes. Moreover, some inclusion criteria differ between studies. In order to partially overcome this limitation, we decided to add age and both depressive and manic subsyndromal symptoms as covariates in the statistical models. Second, the cross-sectional design of this study did not enable us to determine causal inferences between EI, clinical symptomatology, and neurocognition, nor to examine the changes in EI ability associated with neuroprogression in BD. Since the FEM sample size was derived from a longitudinal study, we will be able to provide insight on the course of EI in the early phases of BD, for the patients included in the present study, as soon as the follow-up will be ended. Similarly, the description of influence of treatment should be further detailed. Also, the ability of MSCEIT test to discriminate individuals at the mean and high level of EI has been questioned (Fiori et al., 2014).

Despite these limitations, the strength of the present study is to provide insight on EI in patients in the early stage of the illness, an almost unexplored aspect in this group of patients and is the first investigation aimed at understanding which socio-demographic, clinical, and neurocognitive factors may contribute to EI levels in the early stages of BD. Furthermore, the present study can rely on a quite big sample size for both FEM and BD patients, allowing for a cross-sectional comparison of the EI abilities in two different phases of BD using the four branches of MSCEIT. In particular, BD patients have difficulties in EI but not patients that experienced their FEM over last 3 years. Therefore, our findings suggest that EI is preserved in early stages, which represents an optimistic result. However, this might worsen in later stages of the disease. Difficulties in EI performance might be possibly associated with the increasing burden of disease, and neuroprogression in chronic BD, although this hypothesis will need to be confirmed in longitudinal studies. On the contrary, neurocognition and psychosocial functioning seemed to be impaired at an earlier stage than EI. These findings have important implications in terms of early interventions, which should address not only neurocognitive performance but also social cognitive functioning at the early stages in order to prevent or mitigate the cognitive decline often associated with BD in the long-term (Vieta et al., 2018). Both EI and neurocognitive performance should be assessed in the early stages of the disease. While neurocognitive performance could be already impaired in the early stages and thus represents a target of secondary preventive intervention, EI could be not impaired in the early stages of the disease and should be addressed with primary preventive interventions aimed at possibly avoiding EI difficulties in these patients.

## Data Availability

The data that support the findings of this study are available on request from the corresponding authors.

## Supplementary material

For supplementary material accompanying this paper visit https://doi.org/10.1017/S0033291721005122. click here to view supplementary material
