# Supplementary material for: Emotional intelligence: a comparison between patients after first episode mania and those suffering from chronic bipolar disorder type I
Source: Psychol Med. 2022 Jan 7;53(7):3065–76. doi: 10.1017/S0033291721005122 (PMC10235671; doi:10.1017/S0033291721005122)
Supplement: Supplementary file 1 [file S0033291721005122sup001.docx]

**Supplemental Data**

**Methods**

*Participants*

Subjects with a first episode mania (FEM) were drawn from the “Prodromes and Predictors in First Episode Mania and Psychosis” – ProPreF project. a two-year longitudinal. multicentric study investigating prodromes and predictors of clinical and longitudinal outcomes in patients presenting a FEM or a first episode psychosis (FEP).

*Procedures*

Socio-demographic data, among others age, educational level, working status, were collected and stored in an electronic data repository. Medical records were assessed for completeness of information.

To verify the diagnosis and to determine the presence of a first full psychotic or manic episode, the summaries of the patients’ files, the life charts of psychotic and mood episodes and the assessment of the clinical presentation at first inpatient hospitalization or first mental health service presentation, were reviewed by at least two psychiatrists and an agreement was reached on the diagnosis. If the patient met the DSM-5 A-D criteria for a manic episode a diagnosis of FEM was posed. If the patient presented at least two of the five symptoms of the criterion A for a DSM-5 psychotic disorder and no mood episode co-occurred a diagnosis of FEP was posed. After full or partial clinical remission (i.e. after discharge from the hospital) the patients were clinically assessed by a trained psychiatrist by means of the Structured Clinical Interview for DSM Disorders (SCID-I-II) (First, M., Gibbon, M., Spitzer, R., Williams, J., & Benjamin, 1997a, 1997b) and diagnoses were determined according to DSM-5 criteria. Patients with schizophrenia or a schizophreniform disorder diagnosis were excluded and patients who met bipolar disorder (BD) diagnostic criteria were classified as FEM.

Also HC underwent a semi-structured interview based on the Structured Clinical Interview for DSM Disorders (SCID-I-II) (First, M., Gibbon, M., Spitzer, R., Williams, J., & Benjamin, 1997a, 1997b) to exclude current or past psychiatric history**~~.~~**

*Clinical assessment*

Clinical information was collected for the subgroups of patients, assessing onset features (i.e. age at onset. age at first hospitalization) , characteristics of the longitudinal course (i.e. total number of episodes, number of manic, hippomaniac, depressive and missed episodes, total number of hospitalizations. duration of illness) or the presence of a positive family history for Depressive and Bipolar Disorders and pharmacological treatment (all patients were under stable treatment regimen).

In order to explore the variables associated with the EI performance in patients with a FEM, other specific clinical variables were collected. Particularly, the Premorbid adjustment, namely levels of functioning before the onset of illness,. was assessed with The Premorbid Adjustment Scale (PAS)(Cannon-Spoor, Potkin, & Jed Wyatt, 1982). Only childhood and early adolescence life periods have been taken into account since they are the two periods answered by all the participants. Higher scores indicate worse premorbid adjustment. In addition, information on the presence of psychotic symptoms at onset, the use of alcohol or cannabis before the onset. was also assessed.

*Neurocognitive domains*

Patients’ raw scores on neuropsychological tests were standardized to z-scores (i.e.. M=0. SD=1) based on HCs’ scores using the formula: (test score – HC test M)/HC test SD. Furthermore, several *z*‐scores of different tests were summed and averaged to create six cognitive composites. Following this procedure, cognitive composites were standardized against the composite scores obtained for the HC subgroup. The variables included in each cognitive domain were adjusted to cognitive domains proposed by the ISBD‐BANC (Yatham et al., 2010) as follows: Processing Speed (WAIS-III Digit-symbol Coding, the Category fluency (Animal naming) ,. and the TMT-A); (ii) the Working Memory (WAIS-III (Letter-number sequencing and the Digit-span subtests)); (iii) Verbal Memory (CVLT (total trials 1–5 list A,short free recall, short cued recall, delayed free recall. and delayed cued recall) (iv) Visual Memory (ROCF immediate recall); (v) Executive Functions (WCST (number of categories and perseverative errors). the Stroop Test (Interference) , and the TMT-B); and (vi) Attention (CPT-II (omission, reaction time and reaction time standard error). Outlying z-scores of > 4 SDs below HC mean were then truncated at z = -4.0. The z-scores for CPT-II, WCST perseverative errors, and TMT (A and B) were inverted so that higher scores represented poorer performance.

**Online references**

Cannon-Spoor, H. E., Potkin, S. G., & Jed Wyatt, R. (1982). Measurement of premorbid adjustment in chronic schizophrenia. *Schizophrenia Bulletin*, *8*(3), 470-480. https://doi.org/10.1093/schbul/8.3.470

Fairbairn, J. W., & Rowan, M. G. (1975). Proceedings: Cannabinoid pattern in Cannabis sativa L. seedlings as an indication of chemical race. *The Journal of pharmacy and pharmacology*, *27 Suppl?*-, 90P. Recuperado de http://www.ncbi.nlm.nih.gov/pubmed/2769

First, M., Gibbon, M., Spitzer, R., Williams, J., & Benjamin, L. (1997a). Structured Clinical Interview for DSM-IV Axis I Disorders-Clinician (SCID-I). *American Psychiatric Press: Washington, DC, USA*.

First, M., Gibbon, M., Spitzer, R., Williams, J., & Benjamin, L. (1997b). Structured Clinical Interview for DSM-IV Axis II Personality Disorders (SCID-II). *American Psychiatric Press: Washington, DC, USA*.

Mohammadkhani, P., Jokar, M., Jahani-tabesh, O., & Tamannaei-far, S. (2011). Structured Clinical Interview for DSM-IV Axis II personality disorders (Persian Version). *American Psychiatric Press, Inc.*

Yatham, L. N., Torres, I. J., Malhi, G. S., Frangou, S., Glahn, D. C., Bearden, C. E., … Chengappa, K. N. R. (2010). The International Society for Bipolar Disorders-Battery for Assessment of Neurocognition (ISBD-BANC). *Bipolar Disorders*, *12*(4), 351-363. https://doi.org/10.1111/j.1399-5618.2010.00830.x

**Supplementary tables**

| **Supplementary Table 1. MSCEIT and Neuropsychological Scores of First Episode Mania (FEM) or Bipolar Disorder (BD) patients and Healthy Controls (HC)** | | | | | | | | | | | | | | |  |  |  |  |
| --- | --- | --- | --- | --- | --- | --- | --- | --- | --- | --- | --- | --- | --- | --- | --- | --- | --- | --- |
| **Variables** | **FEM (A)**  **(n=48, 26.09%)**  **Mean**  **(IC 95%)** | | **BD (B)**  **(n=75, 40.76%)**  **Mean**  **(IC 95%)** | | **HC (C)**  **(n=61, 33.15%)**  **Mean**  **(IC 95%)** | **Statistics** | | | | | | | | |  |  |  |  |
|  |  |  |  |  |  | **χ^2^** | | **p** | **Pairwise Comparison*** | | **p**† | **Effect Size**  (Glass’s delta) | | | |  |  |  |
| **Emotional Intelligence** |  |  | |  |  |  |  | | |  |  | |  | | |  |  |  |
| EIQ |  | 114.42  (109.19 – 119.66) | | 107.02  (103.01 – 111.03) | 117.11  (112.80 – 121.43) | 10.748 | **0.005** | | | B<C | **0.004** | | | **0.91** | | |  |  |
| Experiential EI |  | 109.04  (104.25 – 113.82) | | 102.72  (99.06 – 106.39) | 105.59  (101.65 – 109.54) | 3.681 | 0.159 | | |  |  | |  | | |  |  |  |
| Strategic EI |  | 104.92  (100.68 – 109.17) | | 100.31  (97.05 – 103.56) | 105.18  (101.68 – 108.68) | 4.293 | 0.117 | | |  |  | |  | | |  |  |  |
| Perceiving emotions |  | 108.95  (104.18 – 113.72) | | 103.47  (99.81 – 107.13) | 105.01  (101.07 – 108.95) | 2.830 | 0.243 | | |  |  | |  | | |  |  |  |
| Using emotions |  | 105.42  (100.65 – 110.19) | | 100.72  (97.07 – 104.38) | 103.90  (99.97 – 107.84) | 2.308 | 0.315 | | |  |  | |  | | |  |  |  |
| Understanding emotions |  | 104.08  (99.86 – 108.31) | | 97.24  (94.01 – 100.48) | 104.70  (101.22 – 108.19) | 9.955 | **0.007** | | | B<C  B<A | **0.010**  0.056 | | | **0.73**  0.44 | | |  |  |
| Managing emotions |  | 102.05  (97.22 – 106.89) | | 103.35  (99.65 – 107.05) | 103.86  (99.87 – 107.85) | 0.308 | 0.857 | | |  |  | |  | | |  |  |  |
|  |  |  | |  |  |  |  | | |  |  | |  | | |  |  |  |
| **Neurocognition** |  |  | |  |  |  |  | | |  |  | |  | | |  |  |  |
| Processing Speed |  | -1.12  (-1.48 – -0.76) | | -2.09  (-2.37 – -1.82) | -0.16  (-0.45 – 0.13) | 80.454 | **<0.001** | | | A<C  B<C  B<A | **<0.001**  **<0.001**  **<0.001** | | **0.92**  **2.52**  **1.30** | | |  |  |  |
| Verbal Memory |  | -0.66  (-1.06 – -0.26) | | -1.10  (-1.40 – -0.79) | -0.09  (-0.42 – -0.23) | 17.828 | **<0.001** | | | B<C | **<0.001** | | **1.27** | | |  |  |  |
| Working Memory |  | -0.49  (-0.79 – -0.19) | | -0.77  (-1.00 – -0.55) | -0.05  (-0.29 – 0.20) | 16.675 | **<0.001** | | | B<C | **<0.001** | | **0.85** | | |  |  |  |
| Executive Functions |  | -0.96  (-1.40 – -0.52) | | -1.98  (-2.31 – -1.64) | -0.13  (-0.49 – 0.23) | 49.356 | **<0.001** | | | A<C  B<C  B<A | **0.015**  **<0.001**  **0.002** | | **0.71**  **2.21**  **1.08** | | |  |  |  |
| Visual Memory |  | -0.40  (-0.73 – -0.07) | | -0.55  (-0.84 – -0.26) | 0.04  (-0.29 – 0.22) | 6.852 | **0.033** | | | B<C | **0.035** | | **0.81** | | |  |  |  |
| Attention |  | -1.03  (-1.43 – -0.63) | | -2.82  (-3.11 – -2.52) | -0.01  (-0.31 – 0.29) | 168.426 | **<0.001** | | | A<C  B<C  B<A | **<0.001**  **<0.001**  **<0.001** | | **0.95**  **2.87**  **1.25** | | |  |  |  |
| Abbreviations: **EI**=Emotional Intelligence; **EIQ**=Emotional Intelligence Quotient; **IC 95%=**Lower–Upper values within Wald Confidence Interval of 95%; **MSCEIT**=Mayer-Salovey-Caruso Emotional Intelligence Test;  *Only statistically significant or almost significant comparisons are reported. **Bold** for statistically significant values  †Bonferroni post-hoc significance | | | | | | | | | | | | |  | | | | | |

| **Supplementary Table 2.** **Correlations between MSCEIT Emotional Intelligence Quotient (EIQ) and socio-demographic and clinical variables in chronic Bipolar Disorder (BD) patients** | | | | |
| --- | --- | --- | --- | --- |
| **Variables** | **MSCEIT EIQ** | | | |
|  |  | **Mean (SD)** | **Statistics** | |
|  |  |  | **Pearson correlation or Student t** | **p** |
| **Socio-demographic variables** |  |  |  |  |
| Age |  |  | -0.034 | 0.769 |
| Sex | M | 103.50 (16.71) | 0.996 | 0.322 |
|  | F | 107.27 (15.59) |  |  |
| Estimated IQ |  |  | 0.170 | 0.148 |
| **Clinical variables** |  |  |  |  |
| Family History of BD | Y | 105.65 (19.27) | -0.025 | 0.980 |
|  | N | 105.54 (15.36) |  |  |
| Family History of MDE | Y | 105.08 (17.97) | 0.189 | 0.851 |
|  | N | 105.83 (15.34) |  |  |
| Duration of Illness |  |  | -0.055 | 0.642 |
| Total Number of Episodes |  |  | -0.155 | 0.185 |
| Number of Psychiatric Hospitalizations |  |  | **-0.288** | **0.012** |
| Age at first hospitalization |  |  | 0.241 | 0.061 |
| Psychotic Symptoms at Onset | Y | 101.81 (13.79) | -1.898 | 0.062 |
|  | N | 108.78 (17.46) |  |  |
| HAM-D Total Score^†^ |  |  | **-0.268** | **0.021** |
| YMRS Total Score^†^ |  |  | 0.117 | 0.320 |
| FAST Total Score^†^ |  |  | **-0.298** | **0.010** |
| **Psychotropic Medication** |  |  |  |  |
| Lithium | Y | 104.70 (15.28) | 0.807 | 0.422 |
|  | N | 107.88 (17.59) |  |  |
| Antiepileptics | Y | 106.76 (16.76) | -0.546 | 0.587 |
|  | N | 104.73 (15.43) |  |  |
| Antipsychotics | Y | 104.44 (15.80) | 1.376 | 0.173 |
|  | N | 110.63 (16.51) |  |  |
| Antidepressants | Y | 100.61 (15.04) | **2.202** | **0.031** |
|  | N | 108.83 (15.98) |  |  |
| Benzodiazepines | Y | 106.15 (18.61) | -0.197 | 0.923 |
|  | N | 105.68 (15.62) |  |  |
| **Neurocognitive domains** |  |  |  |  |
| Processing Speed Composite |  |  | **0.407** | **<0.001** |
| Verbal Memory Composite |  |  | **0.386** | **0.001** |
| Working Memory Composite |  |  | 0.160 | 0.171 |
| Executive Functions Composite |  |  | **0.353** | **0.002** |
| Visual Memory Composite |  |  | -0.006 | 0.966 |
| Attention Composite |  |  | **0.274** | **0.017** |
| Abbreviations: **BD**=Bipolar Disorder; **EIQ**=Emotional Intelligence Quotient; **FAST**=Functioning Assessment Short Test; **HAM-D**=Hamilton Depression Rating Scale; **IQ**=Intelligence Quotient; **MDE**=Major Depressive Episode; **SD**=Standard deviation; **YMRS**=Young Mania Rating Scale  **Bold** for statistically significant values  **^†^**At Time of Evaluation | | | | |

| **Supplementary Table 3.**  **Hierarchical multiple linear regression of the socio-demographic, clinical and neuropsychological variables associated with MSCEIT Emotional Intelligence Quotient (EIQ) in chronic Bipolar Disorder (BD) patients** | | | | | | |
| --- | --- | --- | --- | --- | --- | --- |
| **Model** | **MSCEIT EIQ** | | | | | |
|  |  | **ßeta** | **t** | | | **p** |
| **1** | F=6.917, df (1.71), p=0.010 |  | |  |  | |
| FAST Total Score^†^ |  | **-0.298** | | -2.630 | **0.010** | |
| Constant |  |  | | 31.004 | <0.001 | |
| **2** | F=3.907, df (2.70), p=0.025 |  | | 29.723 | <0.001 | |
| FAST Total Score^†^ |  | -0.214 | | -1.491 | 0.140 | |
| HAM-D Total Score^†^ |  | -0.137 | | -0.952 | 0.344 | |
| Constant |  |  | |  |  | |
| **3** | F=3.169, df (3.69), p=0.03 |  | |  |  | |
| FAST Total Score^†^ |  | -0.190 | | -1.317 | 0.192 | |
| HAM-D Total Score^†^ |  | -0.096 | | -0.656 | 0.514 | |
| Antidepressants |  | -0.155 | | -1.274 | 0.207 | |
| Constant |  |  | | 29.881 | <0.001 | |
| 4 | F=3.761, df (4.68), p=0.008 |  | |  |  | |
| FAST Total Score^†^ |  | -0.129 | | -0.907 | 0.368 | |
| HAM-D Total Score^†^ |  | -0.104 | | -0.730 | 0.468 | |
| Antidepressants |  | -0.170 | | -1.432 | 0.157 | |
| Number of Psychiatric Hospitalizations |  | **-0.251** | | -2.233 | **0.029** | |
| Constant |  |  | | 29.650 | <0.001 | |
| **5** | F=4.629, df (5.67), p=0.001 |  | |  |  | |
| FAST Total Score^†^ |  | -0.108 | | -0.784 | .436 | |
| HAM-D Total Score^†^ |  | -0.021 | | -.150 | 0.881 | |
| Antidepressants |  | -0.139 | | -1.212 | 0.230 | |
| Number of Psychiatric Hospitalizations |  | **-0.214** | | -1.968 | **0.053** | |
| Processing Speed Composite* |  | **0.301** | | 2.610 | **0.011** | |
| Constant |  |  | | 28.637 | <0.001 | |
| Abbreviations: **BD**=Bipolar Disorder; **df**=degrees of freedom; **EIQ**=Emotional Intelligence Quotient; **FAST**=Functioning Assessment Short Test; **HAM-D**=Hamilton Depression Rating Scale; **IQ**=Intelligence Quotient; **MDE**=Major Depressive Episode; **SD**=Standard deviation; **YMRS**=Young Mania Rating Scale  **Bold** for statistically significant values  **^†^**At Time of Evaluation  * Among the neurocognitive composites, only the Processing Speed Composite was entered in the regression to avoid multicollinearity and because it was the most correlated with the MSCEIT EIQ. | | | | | | |
